# mEH Tyr113His polymorphism and the risk of ovarian cancer development

**DOI:** 10.1186/1757-2215-6-40

**Published:** 2013-06-06

**Authors:** Jian-Hong Zhong, Zhi-Ming Zhang, Le-Qun Li

**Affiliations:** 1 Department of Surgical Oncology, Tumor Hospital of Guangxi Medical University, He Di Rd. #71, Nanning 530021, P.R. China

**Keywords:** Meta-analysis, Microsomal epoxide hydrolase, Polymorphism, Ovarian cancer

## Abstract

**Background:**

The causes of ovarian cancer are complex and may be influenced by many factors, including polymorphism in the microsomal epoxide hydrolase (mEH) gene. Previous work suggests an association between the Tyr113His mEH polymorphism rs1051740 and susceptibility to ovarian cancer, but the results have been inconsistent.

**Methods:**

PubMed, EMBASE, Google Scholar, and Chinese National Knowledge Infrastructure databases were systematically searched to identify relevant studies. A meta-analysis was performed to examine the association between Tyr113His mEH polymorphism and susceptibility to ovarian cancer. Odds ratios (ORs) and 95% confidence intervals (CIs) were calculated.

**Results:**

Five studies involving 2,566 cases and 2,839 controls were included. Although the polymorphism did not affect ovarian cancer risk in the allelic contrast model (OR = 0.99, 95% CI = 0.83-1.17, *P* = 0.86), the mutant CC genotype was significantly associated with increased risk in the homozygote comparison (OR = 1.20, 95% CI = 1.01-1.43, *P* = 0.04) and recessive genetic models (OR = 1.20, 95% CI = 1.01-1.41, *P* = 0.03). The wild-type TT genotype was not associated with higher or lower ovarian cancer risk in the dominant genetic model (OR = 1.04, 95% CI = 0.83-1.29, *P* = 0.74). These results were robust to sensitivity analysis.

**Conclusions:**

The CC genotype of Tyr113His mEH may confer increased risk of ovarian cancer. These conclusions should be verified in large and well-designed studies.

## Introduction

Ovarian cancer is the leading cause of gynecology-related cancer death, with an estimated 22,240 new cases and 14,030 deaths in the United States alone in 2013 [1]. Ovarian cancer is difficult to diagnose early, so it is crucial to identify risk factors in order to promote prevention. Unfortunately the etiology of ovarian cancer remains unclear. Some epidemiological studies suggest that genetic factors play an important role. Candidate risk factors include the genes encoding insulin receptor substrate 1 [2], Lysyl oxidase G473A [3], murine double minute 2 [4], and progesterone receptor [5].

Another candidate risk factor that has received a lot of attention is the gene encoding microsomal epoxide hydrolase (mEH). This gene is overexpressed in ovarian tissue [6], where it plays a role in estrogen production [7]. The protein is also a phase II metabolic enzyme that plays an important role in the activation and detoxification of exogenous chemicals, as well as in the metabolism of epoxides and endogenous steroids [8,9]. Since epoxides are highly reactive oxidative metabolites, mEH is thought to act on the most toxicologically active forms of drugs and environmental chemicals. Despite its important protective function, its net effects on the body can be complex, since it plays a dual role of procarcinogen detoxifier and activator [10].

Recently, a number of studies have examined the possible association between the Tyr113His mEH (rs1051740) polymorphism and ovarian cancer risk [11-15], but the results have been inconsistent. Since individual case–control studies may fail to detect complicated genetic relationships because of small sample size, we performed a meta-analysis of several published studies to provide a more rigorous test of the effects of mEH polymorphism on ovarian cancer risk.

## Materials and methods

### Search strategy

All clinical and experimental case–control studies of mEH polymorphism and ovarian cancer risk published through January 31, 2013 were identified through systematic searches in PubMed, EMBASE, Google Scholar, and Chinese National Knowledge Infrastructure databases. No language restrictions were imposed. The search terms used were: *mEH; HYL1; EPHX; microsomal epoxide hydrolase;* each of these four terms in combination with *polymorphism, variation, genotype, genetic* or *mutation;* each of the above terms in combination with *ovarian cancer* or *carcinoma of ovary*. Reference lists of relevant articles were also manually searched to identify additional relevant publications.

### Inclusion criteria

A study was included in the meta-analysis if it satisfied the following criteria: (a) it assessed the association between ovarian cancer and the Tyr113His mEH gene polymorphism, (b) it used a case–control design, and (c) it provided sufficient published data for estimating an odds ratio (OR) with a 95% confidence interval (95% CI). In the case of multiple studies based on the same population, we selected the study with the largest number of participants.

### Data extraction

Two authors (JHZ and LQL) independently searched the literature and identified eligible articles based on the inclusion criteria. The following data were extracted: first author’s family name, year of publication, patient ethnicity or country, numbers and genotypes of cases and controls, and Hardy-Weinberg equilibrium (HWE) of controls. Discrepancies were resolved by consensus. Only those studies that met the predetermined inclusion criteria were included.

### Statistical methods

The unadjusted odds ratio (OR) with 95% confidence interval (CI) was used to assess the strength of the association between the Tyr113His mEH polymorphism and ovarian cancer risk based on the genotype frequencies in cases and controls. The potential association of different Tyr113His mEH genotypes with ovarian cancer risk was examined by comparing the C allele with the T allele, comparing homozygous genotypes, and applying recessive and dominant genetic models.

All statistical tests for this meta-analysis were performed using RevMan 5.14 (Cochrane Collaboration) and Stata 11.0 (StataCorp, College Station, USA). Fixed-effect and random-effect models were used to calculate pooled ORs. The statistical significance of the pooled ORs was determined using the Z-test, and P < 0.05 was considered statistically significant. The assumption of heterogeneity was evaluated by applying a chi-squared-based Q-test among the studies. In this approach, the Q value is defined to be identical to the effect size of the chi-squared test. P > 0.10 for the Q-test indicates a lack of heterogeneity and suggests that variability in effect sizes is larger than that expected from chance alone [16]. In these cases, the fixed-effect model was used to calculate a pooled OR for each study. Otherwise, the random-effect model was used to calculate pooled ORs. HWE in the control group was assessed using the asymptotic test, with P < 0.05 considered significant. Small-study bias was assessed by Harbord’s modified test [17]. As much as possible, the meta-analyis was carried out according to the PRISMA guidelines [18].

## Results

### Description of studies

**Figure 1:**
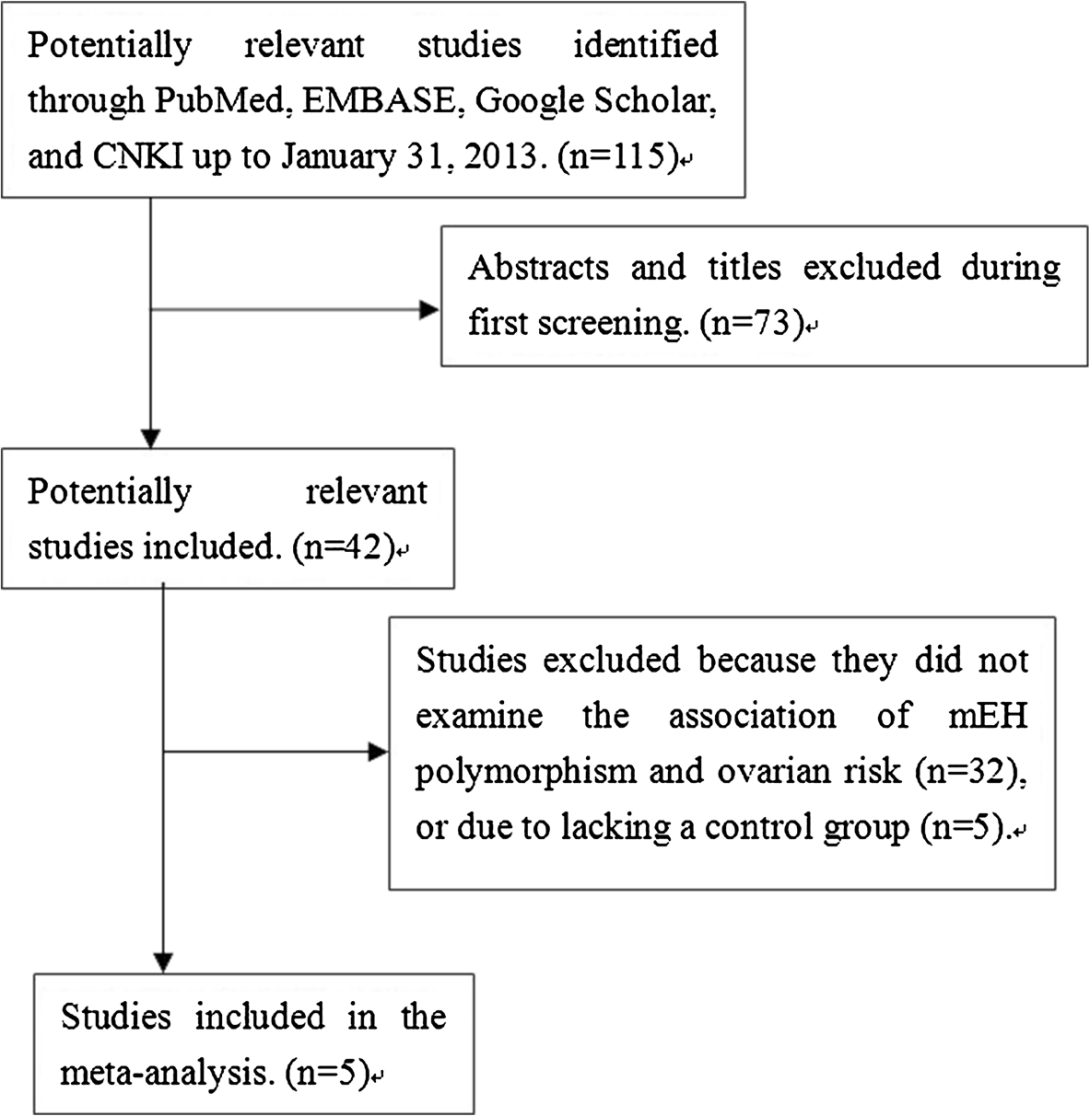
**Flow chart of study selection.** mEH, microsomal epoxide hydrolase.

A total of 115 potentially relevant publications published through January 31, 2013 were systematically identified in the PubMed, EMBASE, Google Scholar, and Chinese National Knowledge Infrastructure databases. Of these, we excluded 32 studies based on review of the titles and abstracts because they did not focus on the association of mEH polymorphism and ovarian cancer risk, or they did not include a control group (n = 5). In the end, only five studies [11-15] involving 2,566 cases and 2,839 controls were found to satisfy the inclusion criteria and included in the meta-analysis (Figure 1). Detailed characteristics of the five studies are listed in Table 1. Participants in four studies [11,12,14,15] were Caucasian, while those in the remaining study [13] were Chinese. Genotype distribution in controls did not show HWE in two studies [12,13]. We failed to find additional eligible studies when we repeated our systematic literature search procedure in May 2013.

**Table 1:** Principal characteristics of studies included in the meta-analysis

**Study**	**Country**	**Ethnicity**	**Genotyping method**	**P**_**HWE**_	**P**_**frequency of T-allele**_	**Cases / Controls**	**No. of cases**			**No. of controls**		
							**TT**	**TC**	**CC**	**TT**	**TC**	**CC**
Baxter 2002^11^	UK	Caucasian	Allele-specific PCR	0.237	0.65	291/257	142	114	35	129	100	28
Goode 2011^12^	US and Australia	Caucasian	Allele-specific PCR	0.034	0.03	1571/2046	767	599	205	1030	815	201
Kang 2004^13^	China	Chinese	Allele-specific PCR	<0.001	0.32	86/174	27	26	33	50	46	78
Lancaster 1996^14^	US	Caucasian	PCR-RFLP	0.626	0.01	73/75	47	17	9	31	33	11
Spurdle 2001^15^	Australia	Caucasian	Allele-specific PCR	0.268	0.63	545/287	255	233	57	142	114	31

One study [12] did not report histological subtype data in detail. However, it provided the OR and 95% CI based on allelic contrast. Three studies [11,13,15] described the histological data in detail, and the remaining study [14] did not mention histological subtype at all.

One of the included studies [11] reported that the polymerase chain reaction restriction fragment length polymorphism (PCR-RFLP) genotyping method could lead to incorrect classification of Tyr/His heterozygotes as His/His homozygotes. Among the five included studies, only one [14] used this approach, whereas the others [11-13,15] used allele-specific PCR. Therefore we conducted sensitivity analysis based on genotyping method to assess the robustness of our meta-analysis results.

### Quantitative data synthesis

**Figure 2:**
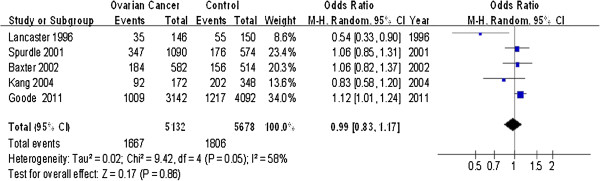
Forest plots describing the association of Tyr113His mEH polymorphism with ovarian cancer (C-allele vs. T-allele).

Table 2 shows the summary ORs for the Tyr113His mEH polymorphism and ovarian cancer risk on the basis of 2,566 cases and 2,839 controls. Calculation of overall OR in the total population using the random-effect model showed that the C allele did not influence risk of ovarian cancer based on allelic contrast (OR = 0.99, 95% CI = 0.83-1.17, *P* = 0.86; *I*^2^ = 58%) (Figure 2). However, the variant CC genotype was associated with increased risk of ovarian cancer relative to the TT genotype (OR = 1.20, 95% CI = 1.01-1.43, *P* = 0.04) and to the TT + TC genotypes (OR = 1.20, 95% CI = 1.01-1.41, *P* = 0.03). In contrast, the wild-type TT genotype was not associated with higher or lower ovarian cancer risk than was the CC + TC genotype (OR = 1.04, 95% CI = 0.83-1.29, *P* = 0.74).

**Table 2:** Overall and stratified meta-analysis of the association between the Tyr113His mEH polymorphism and ovarian cancer risk

**Genotype comparison**	**OR [95% CI]**	**Z (P value)**	**Heterogeneity of study design**			**Analysis model**
			**χ**^**2**^	**df (P value)**	**I**^**2**^	
**Total (2566 cases, 2839 controls)**						
C-allele vs. T-allele	0.99 [0.83, 1.17]	0.17 (0.86)	9.42	4 (0.05)	58%	Random
CC vs. TT	1.20 [1.01, 1.43]	2.05 (0.04)	6.21	4 (0.18)	36%	Fixed
CC vs. TT + TC	1.20 [1.01, 1.41]	2.11 (0.03)	6.04	4 (0.20)	34%	Fixed
TT vs. CC + TC	1.04 [0.83, 1.29]	0.33 (0.74)	9.05	4 (0.06)	56%	Random
**Caucasian (2480 cases, 2665 controls)**						
C-allele vs. T-allele	1.01 [0.85, 1.21]	0.13 (0.89)	7.57	3 (0.06)	60%	Random
CC vs. TT	1.25 [1.04, 1.50]	2.36 (0.02)	4.22	3 (0.24)	29%	Fixed
CC vs. TT + TC	1.26 [1.05, 1.50]	2.56 (0.01)	2.99	3 (0.39)	0%	Fixed
TT vs. CC + TC	1.04 [0.80, 1.34]	0.28 (0.78)	8.73	3 (0.03)	66%	Random
**Chinese (86 cases, 174 controls)**						
C-allele vs. T-allele	0.83 [0.58, 1.20]	0.99 (0.32)				
CC vs. TT	0.78 [0.42, 1.46]	0.77 (0.44)				
CC vs. TT + TC	0.77 [0.45, 1.30]	0.99 (0.32)				
TT vs. CC + TC	1.13 [0.65, 1.99]	0.44 (0.66)				

Small-study bias tests showed no significant bias (*P* = 0.054; Additional file 1: Figure S1).

### Subgroup analysis by ethnicity

#### Chinese ethnicity

After stratification for ethnicity, we observed that in the Chinese population of 86 cases and 174 controls [13], mEH polymorphism was not associated with increased or decreased risk of ovarian cancer based on the C allele, homozygote comparison, or recessive and dominant genetic models (C allele, OR = 0.83, 95% CI = 0.58-1.20, *P* = 0.32; homozygotes, OR = 0.78, 95% CI = 0.42-1.46, *P* = 0.44; recessive model, OR = 0.77, 95% CI = 0.45-1.30, *P* = 0.32; dominant model, OR = 1.13, 95% CI = 0.65-1.99, *P* = 0.66).

#### Caucasian

Analysis of the Caucasian participants in four studies [11,12,14,15] gave results similar to those obtained with the total population (Table 2).

### Subgroup analysis by histological subtype of ovarian cancer

The first subtype analysis was based on three studies with 922 cases and 718 controls [11,13,15]. Meta-analysis showed that individuals with the C allele had significantly reduced risk of endometrioid/clear cell ovarian cancer (OR = 0.54, 95% CI 0.42-0.70, *P* < 0.001). At the same time, the homozygous CC genotype significantly increased the risk of mucinous ovarian cancer relative to the TT genotype (OR = 23.53, 95% CI 9.40-58.93, *P* < 0.001). Significant associations were not detected for comparisons of other genotypes and cancer subtypes (Table 3).

**Table 3:** Association between the Tyr113His mEH polymorphism and risk of ovarian cancer of different histological subtypes

**Genotype comparison**	**OR [95% CI]**	**Z (P value)**	**Heterogeneity of study design**			**Analysis model**
			**χ**^**2**^	**df (P value)**	**I**^**2**^	
**Serous (441 cases, 718 controls)**						
C-allele vs. T-allele	0.87 [0.72, 1.06]	1.39 (0.16)	4.25	2 (0.12)	53%	Fixed
CC vs. TT	0.89 [0.60, 1.33]	0.56 (0.58)	1.85	2 (0.40)	0%	Fixed
CC vs. TT + TC	0.85 [0.59, 1.23]	0.87 (0.38)	1.13	2 (0.57)	0%	Fixed
TT vs. CC + TC	0.99 [0.78, 1.27]	0.06 (0.95)	1.09	2 (0.58)	0%	Fixed
**Endometrioid and clear cell (212 cases, 718 controls)**						
C-allele vs. T-allele	0.54 [0.42, 0.70]	4.67 (<0.001)	0.30	2 (0.86)	0%	Fixed
CC vs. TT	1.30 [0.56, 3.01]	0.62 (0.54)	7.36	2 (0.03)	73%	Random
CC vs. TT + TC	1.33 [0.65, 2.73]	0.78 (0.43)	6.26	2 (0.04)	68%	Random
TT vs. CC + TC	0.88 [0.65, 1.20]	0.78 (0.43)	4.03	2 (0.13)	50%	Fixed
**Mucinous (70 cases, 544 controls)**						
C-allele vs. T-allele	1.08 [0.74, 1.58]	0.40 (0.69)	0.94	1 (0.33)	0%	Fixed
CC vs. TT	23.53 [9.40, 58.93]	6.74 (<0.001)	0.41	1 (0.52)	0%	Fixed
CC vs. TT + TC	0.77 [0.32, 1.86]	0.57 (0.57)	0.13	1 (0.72)	0%	Fixed
TT vs. CC + TC	0.79 [0.48, 1.31]	0.90 (0.37)	0.98	1 (0.32)	0%	Fixed

The second subtype analysis was based on one study involving 1571 cases and 2046 controls [12]. Logistic regression analysis of genotype revealed that the C allele increased serous ovarian cancer risk (OR = 1.17, 95% CI 1.04-1.32, *P* = 0.01). The same allele did not, however, increase or decrease the risk of other types of ovarian cancer.

### Sensitivity analysis

Sensitivity analysis was carried out to assess the robustness of the meta-analysis results to genotyping method, given that one study [14] used PCR-RFLP, which may incorrectly classify Tyr/His heterozygotes as His/His homozygotes [11]. After excluding the PCR-RFLP study [14], meta-analysis showed that risk of ovarian cancer tended to be higher in the presence of the C allele according to allelic contrast (OR = 1.09, 95% CI 1.00-1.18, *P* = 0.06). The CC genotype was associated with increased risk of ovarian cancer both by homozygous comparison (OR = 1.21, 95% CI 1.02-1.43, *P* = 0.03) and by recessive contrast (OR = 1.23, 95% CI 1.03-1.47, *P* = 0.02). In contrast, the TT genotype was not associated with higher or lower ovarian cancer risk based on dominant contrast (OR = 0.94, 95% CI = 0.84-1.05, *P* = 0.29) (Table 4).

**Table 4:** Sensitivity analysis to include only studies using allele-specific PCR as the genotyping method (2493 cases and 2764 controls)

**Genotype comparison**	**OR [95% CI]**	**Z (P value)**	**Heterogeneity of study design**			**Analysis model**
			**χ**^**2**^	**df (P value)**	**I**^**2**^	
C-allele vs. T-allele	1.09 [1.00, 1.18]	1.91 (0.06)	2.44	3 (0.49)	0%	Fixed
CC vs. TT	1.21 [1.02, 1.43]	2.22 (0.03)	5.40	3 (0.14)	44%	Fixed
CC vs. TT + TC	1.23 [1.03, 1.47]	2.30 (0.02)	3.62	3 (0.31)	17%	Fixed
TT vs. CC + TC	0.94 [0.84, 1.05]	1.07 (0.29)	0.53	3 (0.91)	0%	Fixed

## Discussion

Activity of mEH is critical to the metabolism of xenobiotics and procarcinogens that may be involved in initiating cancers such as ovarian cancer. This meta-analysis aimed to evaluate the hypothesis that mEH polymorphism, by altering xenobiotic metabolism, may affect risk of developing ovarian cancer. The results suggest that in the total population and among Caucasians, although not necessarily among Asians, the CC genotype of Tyr113His mEH (rs1051740) is a risk factor of ovarian cancer according to homozygous and recessive contrast. These results were robust to sensitivity analysis in which one study [14] using PCR-RFLP instead of allele-specific PCR was excluded. The available data are insufficient to determine whether particular mEH genotypes affect risk of particular histological subtypes of ovarian cancer.

The Tyr113His mEH polymorphism has been extensively investigated for its potential involvement in various types of cancers, such as colorectal cancer [19], lung and upper digestive tract cancer [20], esophageal carcinoma [21], and hepatocellular carcinoma [22]. Most of these studies, however, have shown that the polymorphism is not a risk factor, except in the case of hepatocellular carcinoma [22]. The present study finds compelling evidence that the homozygous mEH CC genotype may increase ovarian cancer risk. Thus, Tyr113His mEH polymorphism may play an important role in the development of at least two common cancers.

Patients with different histological subtypes of ovarian cancer may have different prognoses [23,24], making it important to understand whether genetic risk applies differently to particular subtypes. Unfortunately this question could not be adequately addressed because most included studies did not report detailed genotype data for different subtypes. Nevertheless, four [11-13,15] of the five included studies reported genotype frequences for some histological subtypes. Among these four studies, two demonstrated that the TT genotype was associated with a decreased risk of endometrioid [15] and serous [11] ovarian cancer. Our meta-analyses based on three studies [11,13,15] involving 922 cases and 718 controls found that the C allele significantly decreased endometrioid/clear cell ovarian cancer risk (Table 3). At the same time, a single study with 1571 cases and 2046 controls [12] showed that the C allele significantly increased serous ovarian cancer risk. Unfortunately the single large study [12] did not describe histological subtype data in detail, so it could not be combined in a meta-analysis with the other three [11,13,15]. Small sample size may explain the discrepancy among the studies. Large, well-designed cohort studies examining the genetic susceptibility of different histological subtypes of ovarian cancer are needed.

The causes of ovarian cancer appear to be complex, involving multiple inherited [25-27], environmental [28,29] and acquired factors [30,31]. While mEH polymorphism may participate directly in influencing risk of ovarian cancer, it may also do so through interactions with other genes or with the environment. For example, one study [20] found cigarette smoking to be associated not only with increased risk of lung cancer but also with increased likelihood of mEH polymorphism. Arguing against this possibility, two studies [12,15] in the present meta-analysis failed to find evidence of synergistic interactions between alcohol or tobacco use and mEH polymorphism on risk of ovarian cancer. The remaining three included studies [11,13,14] did not explore gene-environment or gene-gene interactions. Future studies should examine these interactions in detail, especially given the complexity of ovarian cancer risk factors.

The 113His codon variant is relatively common in the mEH gene, but allele frequency differs significantly among ethnicities. For example, the frequency of the homozygous CC genotype (His113His) is approximately 12% in Caucasians but 40% in Chinese (Table 1). These data are in line with other studies [32,33] showing that the frequency of the His113His variant is greatest in the Asian population (18-42%) and intermediate in the European population (about 10%). Surprisingly, despite its higher frequency in Asians, we did not find any association between the CC genotype or C allele and risk of ovarian cancer in our Chinese ethnic subgroup analysis. In contrast, meta-analysis of all five included studies, with a total participant population that was 95% Caucasian, showed the CC genotype to be a risk factor, and the largest included study [12], involving Caucasians, indicated an association between the C allele and increased risk of serous ovarian cancer. Future work should examine carefully whether the mEH polymorphism affects risk of ovarian cancer differently in different ethnicities. It may be, for example, that the C allele normally increases cancer risk, as observed here in Caucasians, but no longer in Asians because of compensatory mutations with the population could not support such a high frequency of the risk-bearing allele.

The strengths of this meta-analysis include the large number of subjects investigated and the attempt to provide a complete picture taking in account ethnicity, genotyping method and histological subtype of ovarian cancer. However, our review also has several limitations. First, although we included clinical and experimental studies in both English and Chinese in order to avoid local literature bias [34], the number of included studies was quite small. We were unable to increase this number even after systematically searching the databases four months after the original searches. Thus selective publication bias may exist. Second, the results may be affected by additional confounding factors, such as tumor status, age or gender, but most studies either did not report these baseline data or aggregated them in different ways, making it impossible to include them in the meta-analysis. In addition, the vast majority (95.2%) of data may came from Caucasian populations. The numbers of Chinese were relatively small (4.8%). Therefore, the results with Chinese subjects should be interpreted with caution, and future studies on Chinese and African populations should evaluate the ethnicity-specific effects of the mEH polymorphism observed here. In fact, future studies should be more careful about including ethnicity as a confounder variable, since some of the included studies from the US and UK did not report this in detail, potentially leading us to overestimate the proportion of Caucasians in our study population. Finally, although the CC genotype was significantly associated with an increased risk of ovarian cancer compared to the TT genotype or TT + TC genotypes, the 95% CI is near 1, suggesting that the association borders on nonsignificance. Given that the meta-analysis involved more than 5,000 cases and controls altogether, this finding suggests that the effects of mEH risk are likely to be small and modulated by interactions with other genes or the environment.

Implications for future practice and study: This meta-analysis suggests that the mutant CC genotype of Tyr113His mEH may be associated with increased risk of ovarian cancer. However, since this meta-analysis included few studies from non-Caucasian populations, large, well-designed studies in Asian and African populations are warranted to re-evaluate these associations. Our findings also highlight the need for larger, well-designed studies that take into account histological subtype as well as clinically relevant outcomes like overall survival and recurrence rate.

## Abbreviations

HWE: Hardy-weinberg equilibrium of controls; PCR: Polymerase chain reaction; RFLP: Restriction fragment length polymorphism.

## Competing interests

The authors declare that they have no competing interests.

## Authors’ contributions

ZJH contributed to study conception and design. ZJH and ZZM searched the databases and extracted data. ZJH performed all statistical analysis. ZJH and LLQ drafted the manuscript. All authors read and approved the final manuscript.

## Supplementary Material

Additional file 1: Figure S1 Small-study bias tests. Click here for file
