# Supplementary figures and images for: mEH Tyr113His polymorphism and the risk of ovarian cancer development
Source: J Ovarian Res. 2013 Jun 6;6:40. doi: 10.1186/1757-2215-6-40 (PMC3681615; doi:10.1186/1757-2215-6-40)

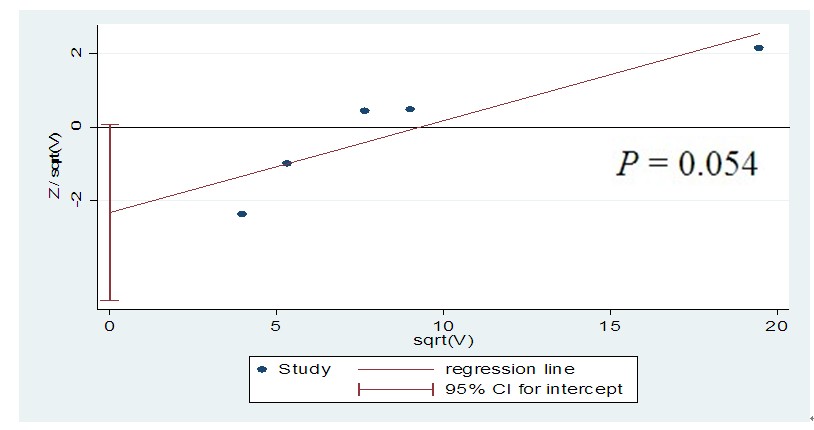

Supplement: Additional file 1: Figure S1 — Small-study bias tests. [file 1757-2215-6-40-S1.jpeg]
